# Road networks and socio-demographic factors to explore COVID-19 infection during its different waves

**DOI:** 10.1038/s41598-024-51610-w

**Published:** 2024-01-18

**Authors:** Shahadat Uddin, Arif Khan, Haohui Lu, Fangyu Zhou, Shakir Karim, Farshid Hajati, Mohammad Ali Moni

**Affiliations:** 1 School of Project Management, Faculty of Engineering, The University of Sydney, Forest Lodge, NSW 2037 Australia; 2 School of Science and Technology, University of New England, Armidale, NSW 2350 Australia; 3 Artificial Intelligence and Cyber Futures Institute, Charles Sturt University, Bathurst, NSW 2795 Australia

**Keywords:** Infectious diseases, Health services

## Abstract

The COVID-19 pandemic triggered an unprecedented level of restrictive measures globally. Most countries resorted to lockdowns at some point to buy the much-needed time for flattening the curve and scaling up vaccination and treatment capacity. Although lockdowns, social distancing and business closures generally slowed the case growth, there is a growing concern about these restrictions' social, economic and psychological impact, especially on the disadvantaged and poorer segments of society. While we are all in this together, these segments often take the heavier toll of the pandemic and face harsher restrictions or get blamed for community transmission. This study proposes a road-network-based networked approach to model mobility patterns between localities during lockdown stages. It utilises a panel regression method to analyse the effects of mobility in transmitting COVID-19 in an Australian context, together with a close look at a suburban population’s characteristics like their age, income and education. Firstly, we attempt to model how the local road networks between the neighbouring suburbs (i.e., neighbourhood measure) and current infection count affect the case growth and how they differ between delta and omicron variants. We use a geographic information system, population and infection data to measure road connections, mobility and transmission probability across the suburbs. We then looked at three socio-demographic variables: age, education and income and explored how they moderate independent and dependent variables (infection rates and neighbourhood measures). The result shows strong model performance to predict infection rate based on neighbourhood road connection. However, apart from age in the delta variant context, the other variables (income and education level) do not seem to moderate the relationship between infection rate and neighbourhood measure. The results indicate that suburbs with a more socio-economically disadvantaged population do not necessarily contribute to more community transmission. The study findings could be potentially helpful for stakeholders in tailoring any health decision for future pandemics.

## Introduction

The COVID-19 pandemic has caused a significant amount of mortality and clinical burden as well as impacted transport, logistics^1^ and economies^2^ globally. Governments put a significant budget and effort into preventing and treating this disease, curbing its growth through public health measures such as mass vaccination, contact tracing, mobility restrictions, lockdowns, etc., and softening the economic loss. In academia, a global effort has been put forward to understand the pathophysical properties of the virus, evaluate public health measures, and model the transmission that could help predict the spread based on historical data.

At the beginning of the pandemic, researchers significantly focused on modelling and predicting the disease transmission and measuring the public health impact. Classical prediction models using the disease spread data have mostly turned out effective. Hernandez-Matamoros et al.^3^ modelled the COVID-19 spread pattern using the autoregressive integrated moving average (ARIMA) model with data from 145 countries, which showed good prediction potential using variables such as population, culture, climate, humidity, etc. Swaraj et al.^4^ proposed an ARIMA-based model that could capture the linear and non-linear components of the disease spread data by integrating an autoregressive neural network. The hybrid method outperformed the single ARIMA model for daily observed cases. Some variations of the classical SIR (Susceptible-Infected-Recovery) model were also used. For example, Abdy et al.^5^ proposed a new SIR model with fuzzy parameters like infection rate, recovery rate, and death rate due to COVID-19. Liu et al.^6^ extended the current susceptible-exposed-infected-recovery (SEIR) model, which is a variation of the SIR model, by incorporating extra compartments. This model can explain the new features of COVID-19 and fine-tune the new model with a neural network aimed at a higher accuracy prediction.

Machine learning models, notably artificial neural networks (ANNs) and recurrent neural networks (RNNs), were preferred when the datasets were much more complicated with more complex features. Car et al.^7^ proposed the first ANN-based model to predict the COVID-19 spread trend. They trained three distinct models using confirmed, recovered and deceased cases and achieved 0.94 for the coefficient of determination. Melin et al.^8^ presented a multiple-ensemble ANN model using a fuzzy response aggregation for time series data. The ensemble ANN models make it possible to predict various conditions, and fuzzy logic could help aggregate the responses of these neural predictors. Beyond these, the best determination coefficient achieved so far is from the experiments by Pinter et al.^9^, who used ANFIS and MLP-ICA methods to predict the number of infected people and the mortality rates. Their determination coefficient score reached 0.99 when applying the MLP-ICA method. The typical modelling using RNN and the best results among RNN variants are developed from the long short-term memory (LSTM) method. Chimmula and Zhang^10^ used an LSTM-based approach to forecast COVID-19 patterns and concluded that the pandemic would come to an end by the end of June 2020. Such a conclusion could be considered quite plausible only for the COVID-19 first wave. Yudistira^11^ also used LSTM to understand and model the correlation of the COVID-19 growth rate. The optimal structure of the models was determined heuristically. Their experiments concluded that LSTM outperformed RNN when using RMSE value as the comparing metrics.

One fundamental premise for the COVID-19 transmission model is that accelerated human mobility increases disease transmission; therefore, most governments employ some mobility restrictions^12^. However, there has been tremendous public debate and concerns for these restrictions' efficacy, reasoning, timeframe and coverage since they significantly impacted different societal groups' quality of life and economic conditions. Although such restrictions have been used during earlier epidemics, the current COVID-19 pandemic is notably different due to its high transmissibility and frequent mutations^13^. A few years after the COVID-19 outbreak, there have been various studies to understand the efficacy of mobility restrictions and business closures and also whether there could be other factors (e.g., income level, economic support, awareness, education, etc.) if improved, could be more effective than mobility restriction to fight the virus. Oh et al.^14^ used Google mobility data and regression models and found that mild and moderate mobility restrictions reduced COVID-19 case counts in most countries. However, severe mobility restriction did not give a proportionately significant case count decrease. Bonaccorsi et al.^15^ used a graph network approach utilising Italian mobility data from Facebook. They highlighted the social costs of lockdown as the mobility restriction has hugely reduced fiscal revenues and increased poverty. Bharati and Fakir^16^ found that stricter rules successfully contained the contagion. However, they also found that restrictions reduced mobility more in relatively less-developed countries. The causal effect of a reduction in mobility on case count was higher in more developed countries. Other similar research^17–19^ also used variations of regression models and found that mobility restrictions at local and international levels have aided in controlling the initial spread of COVID-19. While these studies generally agree that lockdowns were mostly effective in throttling initial spread at the cost of enormous economic cost that affects different socio-economic groups differently, there is still a gap in the applicability of the data sources and the context of different variants of the COVID-19 virus. For example, many studies rely on mobility data provided by third parties which might have sampling bias or specific to certain user groups and can only be observed after the event has been happened. Also, little research has focused on understanding how socio-economic factors moderate mobility restriction and case count in different phases of the COVID-19 pandemic, i.e., during different variant outbreaks.

In this study, we propose a road-network-based network approach to model mobility patterns between localities during lockdown stages and utilise a panel regression method to analyse the effects of mobility in transmitting COVID-19 in the Australian context, together with a close look at a suburban population’s characteristics like their age, income and education. The suburban road network is planned according to local transport demand and, therefore, in an efficient transport system—road connections represent the mobility pattern of the area’s population and could potentially be utilised in disease modelling^20–22^. In the context of the infectious nature of COVID-19, this study adopts a network approach to model the virus's spread within geographic areas, emphasising attributes pertinent to direct viral transmission between individuals. Acknowledging the propensity for increased infections in areas already harbouring infected residents or those connected by roads to high-infection locales, we employ two time-series measures, the prior infection count and a composite measure predicated on the suburban road network, to model the infection numbers in the given suburbs or postal areas.

## Our approach

As summarised in the Introduction section, researchers have used various attributes to model the number of COVID-19 infections for a geographic area in a given period. Given the highly infectious nature of COVID-19, this study considered features that affect the direct transmission of the virus between individuals. There is a good chance of a higher number of future COVID-19 infections in a suburb if it already has an increased number of infected residents. Similarly, the possibility of the same suburb having more infected patients will increase if it has direct road connections with suburbs with many COVID-19-infected patients. Controlling human mobility is challenging at the inter-suburban level; even strict lockdowns or curfews will be in place^23,24^.

Accordingly, this study considered two time-series measures to model the COVID-19 infection number for a given postal area or suburb. The first one is the infection number or count from previous time points. The second is a composite one based on the suburban road network. It is a weighted sum based on the number of road connections to each neighbouring suburb (i.e., the weighting factor) and their respective infection count at the previous time point. The following formula can capture our approach.

1 $$InfNum_{t} = f\left( {InfNum_{{\left( {t - 1} \right)}} , RNInf_{{\left( {t - 1} \right)}} } \right)$$

where $InfNum_{t}$ is the number of infected COVID-19 patients in a suburb at time *t* (i.e., current infection number)*,* $InfNum_{{\left( {t - 1} \right)}}$ is the number of infected COVID-19 patients at time (*t *− *1*) (i.e., previous infection number), and $RNInf_{{\left( {t - 1} \right)}}$ is the road network-based infection measure at (*t *− *1*) (i.e., neighbourhood measure). Mathematically, the following formula represents this measure.

2 $$RNInf_{{\left( {t - 1} \right)}} = \mathop \sum \limits_{i = 1}^{n} \left( {C^{i} \times NorInf_{{\left( {t - 1} \right)}}^{i} } \right)$$

where *n* indicates the number of other suburbs that the underlying suburb has road connections with, $C^{i}$ is the number of road connections the suburb has with the suburb $i$, and $NorInf_{{\left( {t - 1} \right)}}^{i}$ is the normalised infection number of suburb $i$ at $\left( {t - 1} \right)$ time point. This study considers the population sizes of the neighbouring suburbs to normalise their respective infection numbers. Since this measure depends on its connection with neighbouring suburbs and their infection number for a given suburb, this study names it the *neighbourhood measure*.

## Methods and materials

### Data source

This study considered the COVID-19 infection data for 100 different suburbs of the Greater Sydney area of New South Wales, Australia^25^. We considered two distinct periods for the infection statistics of these suburbs: one for the delta variant (4 weeks starting from August 24, 2021) and another for the omicron variant (4 weeks beginning on November 17, 2021). The delta variant also spread during the second period. However, we termed this period ‘omicron’ since the omicron variant had already become prevalent in these suburbs since early November 2021^25^. Table 1 details the basic statistics of the infection data considered in this study.

**Table 1:** The basic statistics of the COVID-19 infection data for 100 suburbs considered in this study.

Omicron	Overall	Week 1 (17–23 Nov 2021)	Week 2 (24–30 Nov 2021)	Week 3 (1–7 Dec 2021)	Week 4 (8–15 Dec 2021)
Mean	8.36	2.80	4.57	6.81	19.27
Change (%)	–	–	63%	49%	183%
Standard deviation	13.72	3.96	6.66	9.85	20.81
Sample variance	188.21	15.68	44.41	97.04	433.03
Minimum	0	0	0	0	0
Maximum	104	24	43	65	104

Delta	Overall	Week 1 (24–30 Aug 2021)	Week 2 (Aug 31–Sep 6, 2021)	Week 3 (7–13 Sep 2021)	Week 4 (14–20 Sep 2021)
Mean	49.69	63.36	55.43	45.15	36.48
Change (%)	–	–	− 13%	− 19%	− 19%
Standard deviation	63.54	77.15	65.78	58.87	46.89
Sample variance	4037.19	5951.93	4327.12	3465.48	2198.80
Minimum	0	0	0	0	0
Maximum	439	439	372	347	239

**Figure 1:**
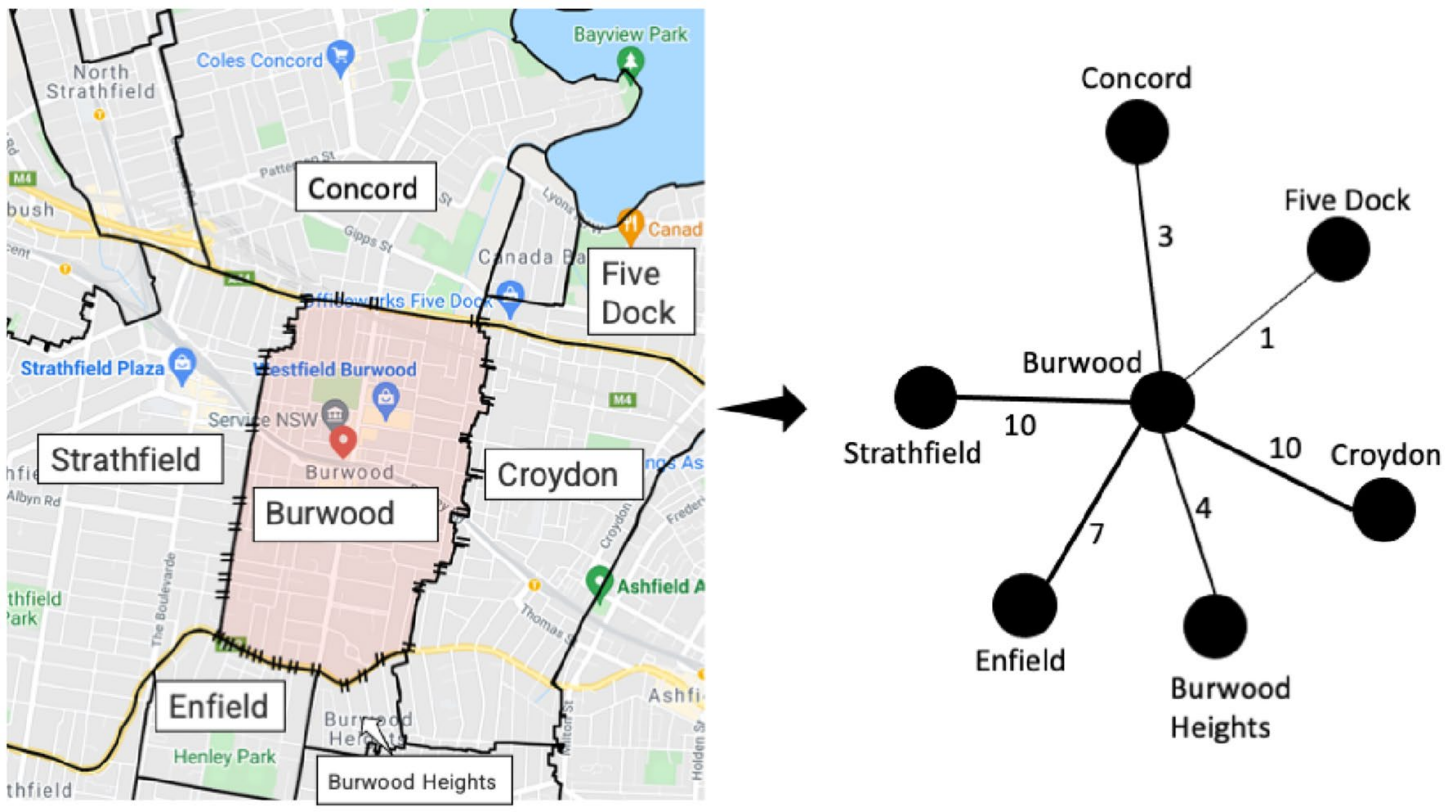
An illustration of the construction of the suburban road network. The left-hand figure shows the map from the Google Maps website. The right-hand figure is the corresponding suburban road network. Burwood (shaded with a light red colour) is the suburb under consideration. Edge weights between two suburbs are the number of roads connecting them. For example, the edge weight (right-hand figure) between Burwood and Strathfield is ten since ten roads connect these two suburbs (left-hand figure). The edge thickness in the right-hand figure proportionates to the corresponding edge weight.

To quantify the second independent variable ($RNInf_{{\left( {t - 1} \right)}}$), we first construct the suburban road network. A node in this network represents a suburb. An edge between two nodes indicates at least one road connecting the underlying suburbs represented by those nodes, and the edge weight points to the number of roads connecting the two suburbs of the edge. We took the map data from Google Maps, Australia^26^. Figure 1 illustrates an example of the suburban road network construction. For a given suburb, we then considered the infection number for each of its neighbouring suburbs. The methodology illustrated in Fig. 1 utilises edge weights to signify the number of roads interlinking suburbs. It is essential to make clear that this method is not saying that the more roads there are between suburbs, like Burwood and Strathfield, the more people will travel between them. Instead, we are using this method as a structured way to estimate possible movement and interactions between different suburbs, serving as a helpful indicator to measure ease of access and connection between areas. These are paramount factors in analysing the potential for virus transmission. By providing a quantitative approximation of interaction and mobility potential, it contributes nuanced insights to understanding the multifaceted dynamics of virus spread. Finally, we used formula (2) to quantify this measure.

This study considered three moderating attributes (i.e., age, education and income) to investigate their impact on the relationship between the dependent and independent variables of this study’s proposed model. The relevant data of these two socio-demographic attributes for different suburbs were collected from the census data provided by the Australian Bureau of Statistics^27^.

### Data analysis design

Since this study repeatedly measured the model variables at four different time points, we followed the panel regression, a powerful tool for modelling time series data^28^, to explore the proposed model. This study used a 1-week duration for each repeated measure. In total, we considered four 1-week windows for the panel regression modelling. We used fixed effect panel regression for research data analysis since we found a significant correlation between the dependent and the independent variables from the initial data exploration. The dependent ${InfNum}_{t}$ variable is significantly correlated at *p < 0.001* with the independent ${InfNum}_{(t-1)}$ variable for delta (*rho* = 0.952) and omicron (*rho* = 0.506) variants. It also has a similar statistically significant correlation with ${RNInf}_{\left(t-1\right)}$ *at p* < 0.001 for the delta (*rho* = 0.771) and omicron (*rho* = − 0.161) data. We used Stata to run the fixed effect panel regression^29^.

This study considered the median population age value, the percentage of residents having a university or tertiary degree, and the median weekly household income to measure the three socio-demographic attributes, age, education and income, respectively, for each suburb. The median values for *age*, *education* and *income* attributes for 100 data instances have split the dataset into two groups. For example, the *education* = *0* group includes all suburbs with a lower percentage of residents having a university degree than the median value of all data instances of this study, and vice versa. We first created six more independent variables to check their moderating strength by multiplying each with the first two independent variables (i.e., InfNum_(t − 1)_ and RNInf_(t − 1)_). Then, we reran the panel regressions, including these six newly created independent variables.

## Results

**Figure 2:**
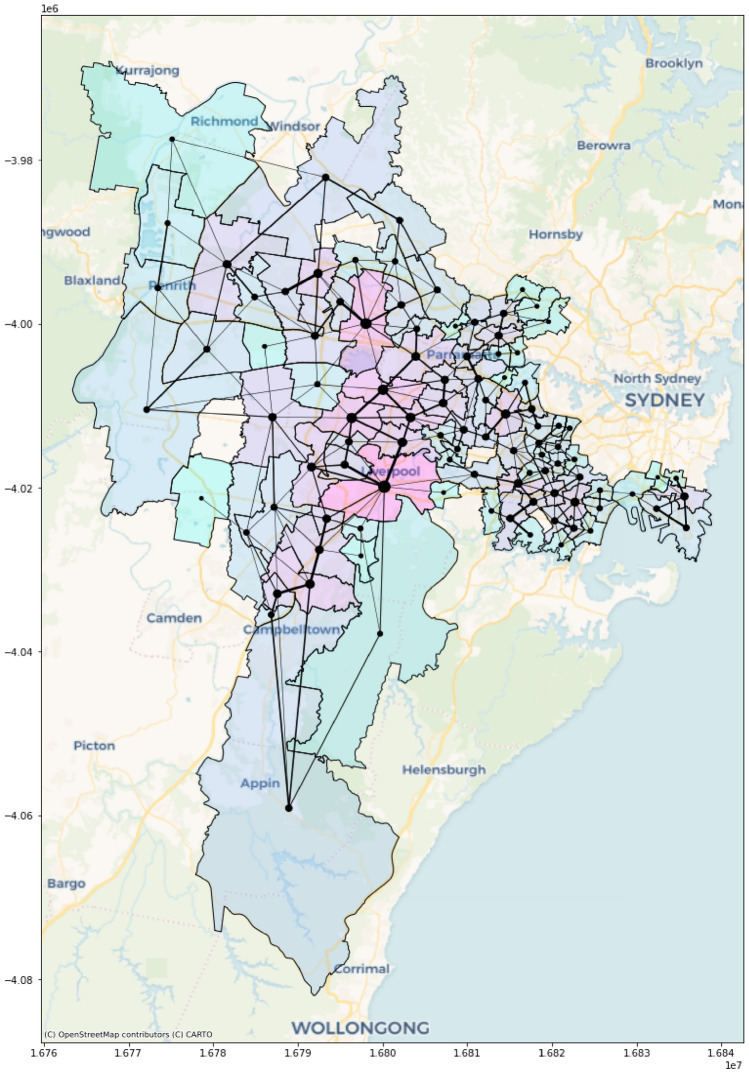
The road network among the 100 suburbs considered in this study. The node's size proportionates to its degree of centrality (i.e., the number of connections it has with its neighbouring suburbs). The edge thickness between two nodes is proportional to the number of roads connecting the corresponding suburbs represented by those two nodes (Map projection: Gephi^30^ and Web Mercator^31^).

Figure 2 illustrates the undirected road network among the 100 suburbs considered in this study. We used Gephi^30^ and web Mercator^31^ projection to draw this road network. In this network, there are 214 undirected edges among its 100 nodes. The maximum number of roads connecting two suburbs is 16, between 2142 and 2160 postal areas.

Table 2 shows the results from the fixed effect panel regressions. The models for both omicron and delta variants show very high R-squared values. The R-squared value for the delta variant is 0.8566, and for the omicron variant, it is 0.5267. The previous infection number (InfNum_(t−1)_) significantly impacts the present infection number for the delta and omicron variants. Neighbourhood measure (RNInf_(t−1)_) also significantly impacts the present infection number. It shows a positive impact on the delta variant. However, it shows a negative effect on the omicron variant.

**Table 2:** Panel regression outcome for delta and omicron variants.

Independent variable	Delta				Omicron			
	Coef	Std. err	t-statistic	Sig	Coef	Std. err	t-statistic	Sig
Constant	8.7439	3.2626	2.68	0.008	6.2191	1.6186	3.84	0.000
InfNum_(t−1)_	0.5466	0.0586	9.33	0.000	1.4319	0.1201	11.92	0.000
RNInf_(t−1)_	0.2642	0.0909	2.91	0.004	− 0.1016	0.0369	− 2.76	0.006
Model parameter								
R-squared	0.8566				0.5267			
F-statistic	96.56				77.38			
Prob (F-statistic)	0.000				0.000			

To check the moderating effect of three socio-demographic attributes (i.e., age, education and income) on the findings of Table 2, we added six more independent variables to our dataset and repeated the same panel regression. These six composite variables are based on multiplying each socio-demographic attribute with the three independent variables. The corresponding results are presented in Table 3. Since our main concern is to check the moderating effect of the three socio-demographic features, we do not report R-squared values in this table. There are no specific patterns revealed in the significance values of this table. The composite independent variables based on the multiplication of *education* and each independent variable do not show any significant outcome for delta and omicron variants. *Age* moderates the relations the present infection number (*InfNum*_*t*_) has with *RNInf*_*(t−1)*_ and *InfNum*_*(t−1)*_ for only the delta variant. For the omicron variant, *age* moderates only the relation between *InfNum*_*(t−1)*_ and *InfNum*_*t*_. Conversely, *income* mediates the association between *InfNum*_*(t−1)*_ and *InfNum*_*t*_ for both variants.

**Table 3:** Panel regression outcome for checking the moderating impact of *age*, *education* and *income.*

Independent variable	Delta				Omicron			
	Coef	Std. err	t-statistic	Sig	Coef	Std. err	t-statistic	Sig
Constant	− 1.0826	0.3731	− 2.90	0.004	0.3318	0.2923	1.14	0.257
InfNum_(t−1)_	0.0906	0.0327	2.77	0.006	− 0.4200	0.0842	− 0.50	0.619
RNInf_(t−1)_	0.3752	0.0864	4.34	0.000	− 0.0710	0.0618	− 1.15	0.252
InfNum_(t−1)_ × age	0.0292	0.0002	174.43	0.000	0.0275	0.0003	107.47	0.000
InfNum_(t−1)_ × education	0.0010	0.0010	0.11	0.916	− 0.0068	0.0035	− 0.19	0.056
InfNum_(t−1)_ × income	0.0000	0.0000	− 2.05	0.041	0.0001	0.0000	2.02	0.044
RNInf_(t−1)_ × age	− 0.0109	0.0023	− 4.80	0.000	0.0023	0.0018	1.31	0.192
RNInf_(t−1)_ × education	− 0.0009	0.0014	− 0.63	0.530	− 0.0001	0.0010	− 0.79	0.427
RNInf_(t−1)_ × Income	0.0000	0.0000	− 0.11	0.910	0.0000	0.0000	− 0.04	0.969

**Figure 3:**
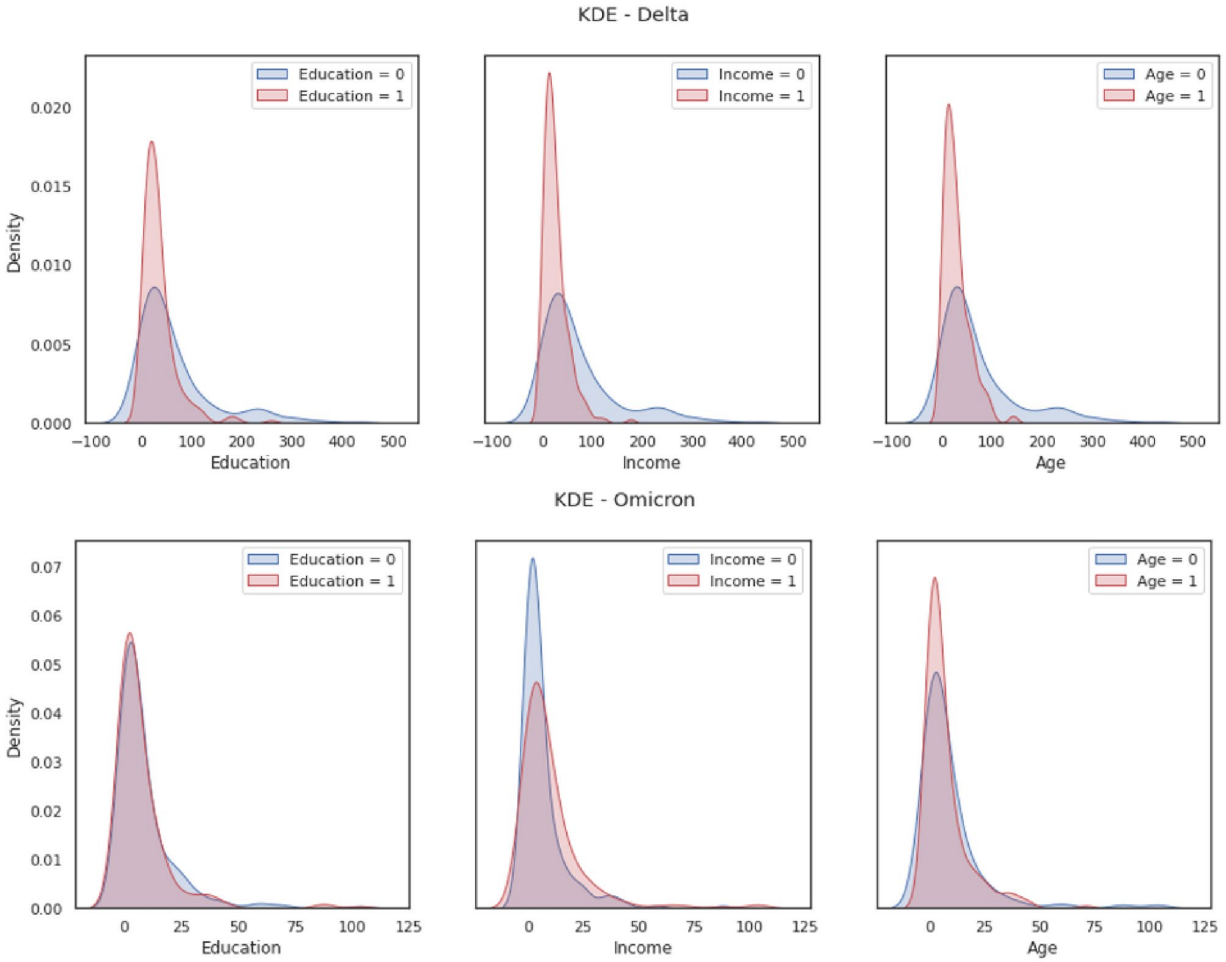
The kernel density estimation of the independent variable (*InfNum*_*t*_) based on the socio-demographic attributes of *age*, *education* and *income.*

Figure 3 shows the kernel density estimation (KDE) for *age*, *education* and *income*. KDE is a non-parametric way to estimate the probability density function of a random variable^32^. The median value of each socio-demographic attribute is used to split the dataset into two groups. The density estimations are based on this study's single dependent variable (*InfNum*_*t*_), divided into two groups by each of the three socio-demographic attributes. This figure reveals that the density functions are closely identical between different groups based on *age*, *education* and *income*, further echoing the findings from Table 3. These three socio-demographic attributes do not reveal specific patterns in moderating the relationship between the model's independent and dependent variables.

## Discussion

Human mobility data has been shown to be an effective measure for modelling COVID-19 infection count^33^. In the first part of this study, we aimed to capture this mobility through the neighbourhood measure and its effect on the COVID-19 infection count. The *neighbourhood measure* considered a relatively granular suburb level as a geographical unit and used the number of shared roads to approximate human movement across the suburbs. The research dataset covers two periods of COVID-19 infection for the delta and omicron variants, as shown in Table 1. One exciting perspective to note and explore in this study is that some of the underlying factors changed between these two timeframes. During the delta outbreak, the research areas were under lockdown (with only allowed shopping limit within a 5 km radius for essential items). Some areas of concern even had nighttime curfew during this timeframe. Sydney’s vaccination coverage (double dose) went from approximately 26–43%^34^. On the other hand, there was no lockdown during the omicron phase of the dataset, although mask mandates, social distancing, and capacity caps in businesses partially remained^35^. Double-dose vaccination coverage (double dose) rose from 77% to almost 79% during this period. As a result, people's mobility within and across the suburbs was inevitably significantly higher during the Omicron outbreak. The omicron variant itself is more transmissible than the delta variant. Therefore, it would be interesting to see how the neighbourhood measure affected the infection count during delta and omicron outbreaks.

The fixed effect panel regression model shows good prediction performance for the delta variant with an R-squared value of 85.66%. The model performance was relatively weaker for the omicron variant, with a 52.67% R-squared value. The previous infection count significantly positively impacts the present infection count (dependent variable) for both variants. The same goes for the neighbourhood measure on its effect on the present infection count, except that for delta, the effect is positive, and for omicron, it is negative. Together, these results indicate that infection counts for a suburb during the delta variant can be well modelled through past infection count and influx from surrounding suburbs, i.e., *neighbourhood measure*. While the present infection count should naturally be affected by the previous infection count, the effect of influx from the neighbourhood is more interesting. As we mentioned earlier, especially during the delta outbreak, there was a lockdown in place, and residents were only allowed to go out for essential shopping within a 5 km radius. Suburbs considered in our research are relatively granular in size, and residents could move across the neighbouring suburbs for essential reasons even while staying within a 5 km bubble. Therefore, this prediction model using suburb-level granular data effectively captures macro-movement during the lockdown and utilises it to predict case counts during the delta variant.

The regression model and the neighbourhood measure did not reveal many insights for the omicron variant because the R-square value was not much higher than the delta variant, and the neighbourhood measure showed a significant negative impact on infection count counter-intuitively. Two factors could contribute to this finding. First, there was no lockdown or movement restriction during the omicron variant. Second, omicron is more transmissible than the delta variant^36^. The high contagiousness and unrestricted movement within the suburb might make the neighbourhood measure less reliable in predicting the case count for omicron.

In the second part of this research, we looked into three socio-economic moderating factors—age, education and income. We intended to see whether suburbs with more residents of higher age brackets, education levels or income differ from suburbs having fewer residents with those factors in terms of case count and neighbourhood measure. This was important in a way that during the delta outbreak, a lockdown was imposed in the areas of concern and a nighttime curfew for some time. These areas of concern were mostly concentrated in western Sydney, where many residents are culturally and linguistically diverse and have a migrant background. These suburbs have more members per household, less income, and education levels on average. Many of the wage earners’ jobs could not be performed from home. Consequently, stay-at-home orders and the lockdown hard hit these suburbs more^37^. Therefore, we investigated these suburbs with high population and COVID-19 cases and explored whether age, education and income have any moderating effect on the case count and neighbourhood measure.

The results in Table 3 summarily show the moderating effects. Education did not have any moderating impact on any combination. Age and income significantly moderated the relation between delta and omicron variants' previous and present case counts. However, income has a small coefficient value for the moderating effect and thus does not reveal any meaningful insight. Age has a positive coefficient, indicating that suburbs with a higher age bracket tended to have higher case growth. This goes along with the fact that older people are at higher risk of comorbidities and COVID-19^38^. Age positively moderates the relation between neighbourhood measure and present case count only for the delta variant. This might indicate that suburbs with a relatively higher aged population tend to have more mobility (for work or essential purposes) if they have more options to travel across suburbs through the higher number of available road connections. For the omicron variant, we have seen earlier that the neighbourhood measure does not affect the case count, probably due to the high transmissibility of the variant and increased local movement due to the absence of lockdown. Consequently, none of the socio-economic variables moderated the relation between the neighbourhood measure and case count.

There are studies in the current literature that explore how restrictions mitigate the adverse COVID-19 effects from various perspectives. Like this study, some used network analysis and statistical modelling^15,19^. As outlined in Table 4, like our study, any mobility restrictions helped reduce COVID-19 negative impacts in one way or another. Our study successfully developed models to explore future COVID-19 infection rates based on prior data and road network density, indicating its uniqueness and novelty. Instead of exploring the direct effect of mobility restriction, our study showed how infection counts could be better estimated, thus controlled, from road network features and previous infection data.

**Table 4:** A comparison of this study and other similar studies from the literature.

Study	Methods used	Findings
Oh et al.^14^	Google mobility data and regression models	Mild to moderate mobility restrictions reduced COVID-19 case counts
Bonaccorsi et al.^15^	Graph network approach	Mobility restrictions hugely reduced revenue and increased poverty
Bharati and Fakir^16^	Heterogeneity analysis using least squares estimation	Stricker rules contained the contagion. Restrictions reduced mobility more in relatively less-developed countries
Sharma et al.^18^	Generalised linear model	Countries with high tourism activity are affected early by COVID-19 restrictions
Li et al.^19^	Network analysis and Statistical models	Road density, population density, and land value positively impact the spread of COVID-19
Uddin et al.^22^	Network analysis and regression	The structure of the suburban road network affects COVID-19 vulnerability and severity
This study	Network analysis and panel regression	Road network density and prior infection rate significantly impact the future COVID-19 infection rate

From the methodological viewpoint, our study faces some limitations, which can potentially create future research scopes. First, we did not consider the number of road lanes connecting two suburbs while capturing road networks. A two-lane road or a multi-lane highway could connect two suburbs, thus representing different transport capabilities. Second, although panel regression is a widely used modelling method for longitudinal data, it has several assumptions on the research dataset used for modelling. In future studies, we aim to explore these assumptions and how they impact model performance by adopting other existing methods (e.g., Bayesian structural time series model^39^) to capture temporal dynamics.

## Conclusion

The Greater Sydney area residents endured nearly 4 months of COVID-19 lockdown during the last half of 2021. While the lockdown bought precious time to ramp up vaccination rollout and prepare healthcare facilities, it left a lasting economic and psychological impact. This study analysed the mobility and prevalence data in two distinct timeframes to model and predict the COVID-19 case count during late 2021. The timeframes represented delta and omicron outbreaks, respectively, and for the former outbreak, there was a lockdown in place and a nighttime curfew for some period. The road network between the neighbouring suburbs was used to approximate the influx and corresponding risk of case growth from adjacent areas. Therefore, this study helps us explore and compare the effect of mobility and case count during and without a lockdown period. It also provides a comparison between delta and omicron variants. The moderating effect of three socio-economic variables is discussed. The method introduced in this study shows an effective way to utilise geographic information and road connection networks with health data to model COVID-19 transmission. The regression model results show that the road network-based neighbourhood measure significantly predicts the case count for the delta variant. The results also show that the income or education level of the residents does not necessarily have any effect in moderating the case count and mobility. The methodology presented in this study could be replicated for other states or countries to gather similar insights.

## Data Availability

The datasets used and/or analysed during the current study are available from the corresponding author upon reasonable request.
